# Role of Signaling Pathways in the Viral Life Cycle 2.0

**DOI:** 10.3390/ijms23147857

**Published:** 2022-07-16

**Authors:** Ugo Moens

**Affiliations:** Molecular Inflammation Research Group, Department of Medical Biology, Faculty of Health Sciences, University of Tromsø—The Arctic University of Norway, 9037 Tromsø, Norway; ugo.moens@uit.no

Viral infections can lead to the generation of new virus particles, whereas other viruses behave as chameleons by camouflaging themselves to evade or mislead the immune system of the host, thereby establishing a latent infection. Yet, other viral infections may cause transformation of the host cell. No matter the outcome of a viral infection, viruses are masters of usurping the host cell’s machinery to facilitate their life cycle. In the case of a productive infection, these pathogens may avail themselves of cellular proteins to enter the host cell, to support the replication and expression of their genome, to produce new, mature virus particles and to be released. Viruses will also utilize cellular proteins to establish a latent or persistent infection, and even to transform the cell. One of the mechanisms by which viruses appropriate the cellular machinery is by manipulating signal transduction pathways, which control numerous cellular processes (Figure 1).

This Special Issue, “Role of Signaling Pathways in the Viral Life Cycle 2.0”, compiles nine contributions; three original articles and six reviews. Three papers describe the interaction of specific signal transduction pathways with the DNA viruses human papillomavirus (HPV), Merkel cell polyomavirus (MCPyV) and hepatitis B virus (HBV), respectively, whereas four manuscripts focus on signaling pathways and the RNA viruses influenza A virus (IAV), severe acute respiratory syndrome coronavirus 2 (SARS-CoV-2), and neurotropic RNA viruses. Finally, two papers discuss the role of nucleoporin RanBP2/Nup358 and tetraspanins in viral infection in general.

Warowicka and colleagues reviewed the role of the transcription factor Yin Yang 1 (YY1) in the life cycle of HPVs and in cancer development [1]. YY1 is a zinc-finger DNA binding protein that can act as either a transcriptional activator or repressor depending on the cellular context [2]. Besides regulating transcription, YY1 plays important roles in other cellular processes such as cell survival, proliferation, and differentiation [2]. YY1 is implicated in carcinogenesis by regulating DNA methylation, cell proliferation, migration and invasion [3]. In undifferentiated keratinocytes, YY1 seems to repress the transcription of the major HPV oncogenes *E6* and *E7* by interacting with YY1-binding sites in the HPV transcriptional control region (LCR) and by mediating epigenetic changes. In differentiated keratinocytes, however, repression is relieved because YY1 levels become reduced. Interestingly, YY1-binding sites in the HPV16 LCR are often mutated in cervical cancer, allowing increased expression of the E6 and E7 oncoproteins. The authors also explored the role of YY1 in virus-independent cancers and in the transcriptional regulation of other human viruses, such as retroviruses, herpesviruses, and HBV [1].

Epigenetic changes such as DNA methylation, histone modifications, chromatin remodeling, and the expression of noncoding RNA are a feature of tumorigenesis [4]. The review by Zhang et al. describes aberrant DNA methylation patterns associated with HBV infection and how this can be involved in HBV-induced hepatocellular carcinoma (HCC) [5]. The viral protein HBx induces host epigenetic changes by, among others mechanisms, increasing the expression and activity of the DNA methyltransferases DNMT1, 3A and 3B, resulting in the selective hyperphosphorylation of the promoters of tumor suppressor genes, genes that are associated with HCC. HBx also affects the expression of cellular microRNAs by promoter methylation changes. By interfering with the gene expression network, HBx inhibits apoptosis, induces epithelial–mesenchymal transition, represses anti-viral immune responses, and stimulates proliferation, known hallmarks of cancer [6].

Another virus associated with cancer is Merkel cell polyomavirus. The virus is the cause of approximately 80% of all Merkel cell carcinomas (MCCs), an aggressive skin cancer [7]. The original paper by Rasheed and coworkers showed that the MCPyV oncoproteins large T- and small t-antigen triggered the expression of interleukin-33, a cytokine linked to increased malignancy and worse prognosis in various types of cancer. These viral proteins induced the activity of the IL-33 promoter and its receptor ST2/IL1RL1. IL-33 activated the NFκB and the mitogen-activated protein kinase (MAPK) pathway ERK. Both IL-33 and its receptor were highly expressed in MCC tumor samples and elevated levels were measured in the plasma of MCC patients compared to healthy controls. Interestingly, IL-33 stimulated the MCPyV promoter activity, resulting in increased expression levels of the viral oncoproteins. Because of the well-documented role of IL-33 in tumorigenesis, the IL-33/ST2 axis may be a therapeutic target for MCC treatment [8]. 

Both the paper by Harris and colleges and the paper by Gerlt and coworkers deal with the respiratory pathogen IAV [9,10]. The host innate immune response is essential to inhibit IAV replication. Harris et al. infected 3-month-old and aged adult mice with mouse-adapted H1N1 and H3N2 IAV. Old mice displayed increased body weight loss, virus titer, cellular infiltration, inflammation, and damage to the alveolar capillary barrier compared to young mice. The authors observed the altered expression of several genes associated with interferon type I signaling and the Toll-like receptor (TLR) and retinoic acid inducible gene-I (RIG-I) pathways in young and aged lung, suggesting perturbed activation of these pathways. Several genes encoding proteins of the MAPK pathway showed diminished upregulation in aged lung compared to young lung in response to IAV infection. Furthermore, differences between H1N1- and H3N2-infected animals were observed. The age- and strain-dependent attenuation of the innate immune responses to IAV infection in mice reflects the phenotypic differences in infected young and old mice and suggests that the decline in innate immunity functions in elderly may contribute to the increased susceptibility to influenza [9]. Gerlt and colleges studied the role of protein phosphatase 2A (PP2A) in IAV replication [10]. By using RNA interference-mediated knockdown of the catalytic subunit of PP2A or the inhibition of PP2A by okadaic acid, the authors found that PP2A exhibited a supportive role in IAV replication in cells by increasing cell survival after viral infection. PP2A exerted this function by modulating the activity of protein kinases involved in apoptotic pathways such as PI3K/Akt, MAPK, JAK/STAT and NFκB. The results indicate that PP2A may form a target for the treatment of IAV infections.

Another respiratory virus that has not been out of the news in the last few years is SARS-CoV-2. In their review, Zhang and Friedman discuss the role of PDZ domain-containing proteins in viral entry and how SARS-CoV-2 can disrupt G-protein coupled receptor (GPCR) signaling [11]. The PDZ domain, an acronym comprising post-synaptic density protein (PSD95), Drosophila disc large tumor suppressor (DLgA), and zonula occludens-1 (ZO-1), consists of 80–90 amino acids and is conserved in the proteins of pro-and eukaryotes [12]. The uptake of SARS-CoV-2 virions is facilitated through the interaction of viral proteins with several PDZ domain-containing proteins. The SARS-CoV-2 spike glycoprotein S is cleaved into S1 and S2, and S1 binds the host cell receptor angiotensin-converting enzyme 2 (ACE2), a PDZ domain-containing protein. The virions also interact with the PDZ domain-containing proteins neuropilin (NRP1) and protein associated with LIN7 1 (PALS1, also known as membrane protein, palmitoylated 5; MPP5), which facilitates the viral penetration of the target cell. The viral E protein binds PALS1. The viral proteins N and 3a contain PDZ-binding motifs, but the PDZ domain-containing proteins that interact with N and 3a have not been characterized, nor is the biological function known. GPCR signaling requires the complex of different proteins that interact with the GPCR to form the GPCR signalosome [13]. PDZ domain-containing proteins can interact with GPCRs and regulate GPCR subcellular localization, endocytosis, trafficking, and signal transduction [14,15]. The Na^+^/H^+^ exchanger regulator factor 1 (NHERF1), a PDZ domain-containing protein, and β-arrestin are components of the GPCR signalosome and can be hijacked by SARS-CoV-2, thereby causing dysfunctional GPCR signaling. β-arrestin can also activate G protein-independent signaling and regulate multiple signaling cascades, include the MAPK pathway. Disrupting interactions between viral proteins and PDZ domain-containing proteins may be a strategy to treat infected patients [11].

The review by Vazquez and Jurado elaborates on mechanisms that different viruses infecting the central nervous system (CNS) use to modulate the immune response [16]. One major strategy implies the regulation of interferon (IFN) secretion by inhibiting components of the pattern recognition receptor-mediated signaling (e.g., TLR3 and TRIF, STAT1, IRF1, IRAK1 and 2) and by inducing autophagy to quench IFNβ production. Other approaches include sequestering host immune proteins from their normal subcellular localization, the modulation of post-translational modification, targeting host immune proteins for cleavage and degradation by viral encoded proteases or by depleting their expression by microRNAs. This diversity of mechanisms illustrates the genius of viruses to deceive their hosts. However, many of the previous studies were conducted in non-CNS cell lines, urging the use of more relevant primary cells or established cell lines.

The review by Jiang and colleges discusses how mutations in the Ran binding protein 2 (RanBP2 or nucleoporin 358 KDa), a component of the nuclear pore complex, interacts with distinct viruses and affects infection [17]. RanBP2 is a multifunctional protein that has sumoylation E3-ligase activity, but also participates in other cellular processes including nucleocytoplasmic transport, the trafficking of photoreceptors, mitosis, energy maintenance, myogenesis, mRNA metabolism, and microRNA-induced silencing [18]. Mutations in RanBP2 are associated with acute-necrotizing encephalopathy type 1 (ANE1), a pediatric neurological disease characterized by the overproduction of cytokines. IAV, parainfluenza virus, human herpes virus 6, respiratory syncytial virus, adenovirus, rhinovirus, rotavirus, and SARS-CoV-2 have all been associated with ANE1. The exact mechanisms by which viruses trigger ANE1 in individuals with RanBP2 mutations remain unclear because viruses can both activate and counteract the action of RanBP2 [17]. ANE-1-associated mutations in RANBP2 were observed to promote viral infection by facilitating viral uncoating, the nuclear import of viral DNA and proteins, and viral assembly. On the other hand, mutations in RanBP2 can hyperactivate the innate immune pathways by stimulating the transcription factors interferon regulator factors 3 and 7, NFκB, and STAT. This will result in the overproduction of anti-viral cytokines. SARS-CoV-2 ORF6 was found to modulate the export of cytokine mRNAs in a RanBP2-dependent manner, but another study reported that SARS-CoV-2 infection reduced RanBP2 expression levels (reviewed in [17]). Further studies are required to disclose the pathogenesis of virus-induced ANE1.

New et al. provide an update on the dual role of tetraspanins, a family of transmembrane glycoproteins, in the life cycle of HPV, IAV, human immunodeficiency virus type 1 (HIV-1), Zika virus (ZIKV), and coronavirus [19]. Viruses avail themselves of tetraspanins at different stages of their life cycle, including receptor attachment to the host cell, endocytosis, trafficking, entry, viral replication, nuclear export, and viral budding. Tetraspanin CD9 is utilized by HPV, HIV-1, and CoVs. CD63 is hijacked by HPV, HIV-1, CoVs, and ZIKV. HIV-1, IAV and CoVs all use CD81, and HPV and IAV usurp CD151. While most tetraspanins are used for viral entry and/or viral egress, some are used to stimulate viral genome replication (e.g., CD63 and CD81 for HIV). Tetraspanin TSPAN7 on dendritic cells fulfils in the function of spreading HIV in the host. This tetraspanin is responsible for the extracellular retention of HIV-1 and as such dendritic cells serve as couriers for HIV-1, which can present virus particles to susceptible CD4+ T lymphocytes. Despite the differences in the amino acid sequences of the viral proteins, the same tetraspanins are recruited, illustrating that viruses apply common mechanisms to replicate. However, tetraspanins can also play a beneficial role for the host by preventing viral infection. Studies have demonstrated that these glycoproteins act in a virus-specific and a cell-specific manner. Although tetraspanins have emerged as attractive therapeutic targets, the use of tetraspanin inhibitors is challenging because of the opposite effect they can have on the viral life cycle.

In conclusion, viruses usurp different signaling pathways to facilitate different stages in their life cycle, but also to contribute to the pathogenicity of viruses. Preventing viruses from taking advantage of signaling pathways may be used to tailor specific antiviral strategies. However, the importance of these pathways for normal host cell function may lead to off-target effects and harm the treated host when signaling cascades are inhibited.

## Figures and Tables

**Figure 1:**
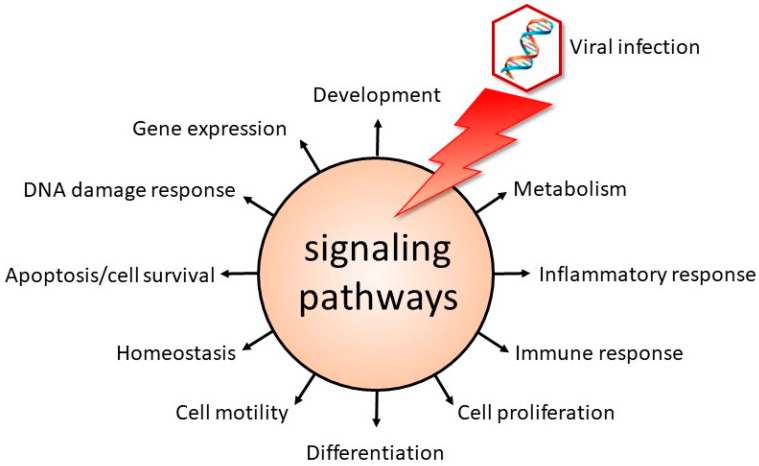
Viruses interfere with signaling pathways to facilitate their life cycle. Virus-mediated usurping of components of signaling cascades will modulate cellular processes. Some of the cellular functions controlled by signal transduction pathways are shown.
